# P-254. Change in Utilization of Statin Therapy Among People with HIV with the Recent Guideline Changes at a Ryan White Clinic in New Orleans, Louisiana

**DOI:** 10.1093/ofid/ofaf695.475

**Published:** 2026-01-11

**Authors:** Tat Yau, Thuong Dien Nguyen-Bui, Lauren Richey

**Affiliations:** LSU Health New Orleans School of Medicine, New Orleans, LA; LSU Health New Orleans, New Orleans, Louisiana; LSU Health Sciences Center New Orleans, New Orleans, Louisiana

## Abstract

**Background:**

Given the promising results of the REPRIEVE trial, the HHS Panel has updated its recommendations for the use of statins as primary prevention against atherosclerotic cardiovascular disease (ASCVD) in people living with HIV (PWH). In particular, the Panel recommends initiating at least moderate-intensity statin therapy for PWH with a 10-year ASCVD risk score of 5% to 20%.

The HIV Outpatient Program (HOP) at the University Medical Center New Orleans (UMCNO) provides care to PWH in the Greater New Orleans metropolitan area. Most patients at HOP are aged between 40 and 75. They could benefit from initiating statin therapy based on the updated guidelines.

**Table 1:**
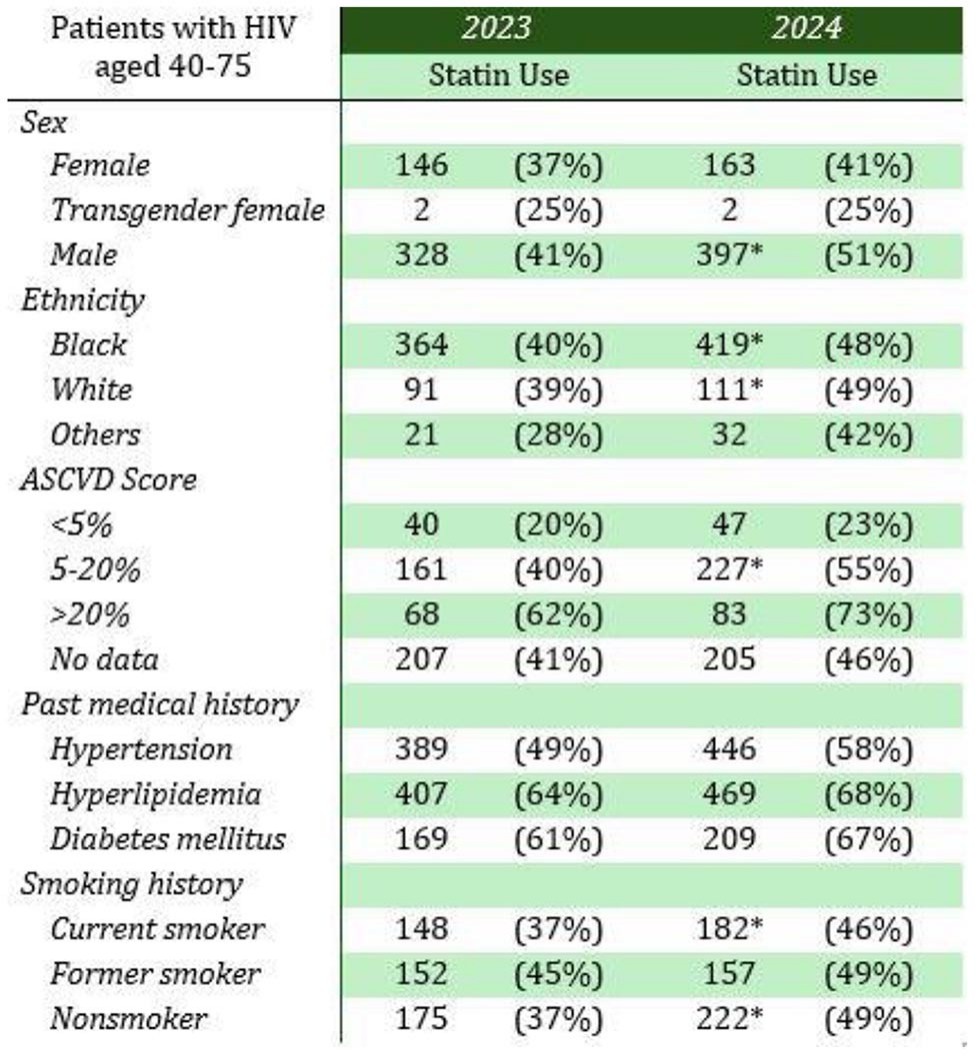
Characteristics of patients with HIV aged 40-75 on statin therapy prior to (2023) and after (2024) the updated guidelines.

**Methods:**

This study aimed to assess compliance with the updated standard of care recommendations for statin therapy. Data was retrieved using Slicer Dicer from Epic Hyperspace. PWH aged 40 to 75 who received care at HOP in the years 2023 and 2024 were evaluated. The analysis included patients' demographics, ASCVD risk scores, relevant past medical history, smoking history, and statin use.

**Results:**

There was a statistically significant increase of 15% in statin use among PWH aged 40 to 75 with an ASCVD score of 5% to 20% (See table 1). Notably, there were statistically significant increases in statin use among men, individuals of black and white race, current smokers, and nonsmokers.

**Conclusion:**

The study demonstrated successful adoption of the updated guidelines on statin utilization among the medical providers at the Ryan White Clinic in New Orleans, Louisiana. However, there is still more room for improvement. Allergies and intolerances could not be accounted for in the data set. Data was not available to calculate the ASCVD for 40% of patients, limiting this analysis.

**Disclosures:**

All Authors: No reported disclosures

